# Supplementary material for: Modeling glioblastoma heterogeneity as a dynamic network of cell states
Source: Mol Syst Biol. 2021 Sep 16;17(9):e10105. doi: 10.15252/msb.202010105 (PMC8444284; doi:10.15252/msb.202010105)
Supplement: Supplementary file 5 — Source Data for Figure 3 [file MSB-17-e10105-s001.zip › Figure3A_sourcedata/GSEA_3065/hallmarks_state6.GseaPreranked.1623416308702/HALLMARK_UV_RESPONSE_UP.html]

Details for gene set HALLMARK\_UV\_RESPONSE\_UP[GSEA]

|  || Dataset | state6 |
| Phenotype | NoPhenotypeAvailable |
| Upregulated in class | na\_pos |
| GeneSet | HALLMARK\_UV\_RESPONSE\_UP |
| Enrichment Score (ES) | 0.25886613 |
| Normalized Enrichment Score (NES) | 1.1287198 |
| Nominal p-value | 0.17983651 |
| FDR q-value | 0.3300283 |
| FWER p-Value | 0.993 |
Table: GSEA Results Summary

  

Fig 1: Enrichment plot: HALLMARK\_UV\_RESPONSE\_UP      
 Profile of the Running ES Score & Positions of GeneSet Members on the Rank Ordered List

  

| PROBE | GENE SYMBOL | GENE\_TITLE | RANK IN GENE LIST | RANK METRIC SCORE | RUNNING ES | CORE ENRICHMENT || 1 | HMOX1 |  |  | 0 | 0.545 | 0.0628 | Yes |
| 2 | BTG1 |  |  | 4 | 0.348 | 0.1024 | Yes |
| 3 | CNP |  |  | 18 | 0.288 | 0.1341 | Yes |
| 4 | BTG2 |  |  | 63 | 0.234 | 0.1561 | Yes |
| 5 | CLTB |  |  | 278 | 0.155 | 0.1493 | Yes |
| 6 | SELENOW |  |  | 297 | 0.152 | 0.1647 | Yes |
| 7 | TGFBRAP1 |  |  | 388 | 0.138 | 0.1702 | Yes |
| 8 | RFC4 |  |  | 397 | 0.136 | 0.1850 | Yes |
| 9 | ATP6V1F |  |  | 407 | 0.135 | 0.1995 | Yes |
| 10 | STK25 |  |  | 412 | 0.134 | 0.2145 | Yes |
| 11 | PPIF |  |  | 465 | 0.127 | 0.2231 | Yes |
| 12 | ATF3 |  |  | 494 | 0.124 | 0.2342 | Yes |
| 13 | CEBPG |  |  | 640 | 0.111 | 0.2302 | Yes |
| 14 | SQSTM1 |  |  | 719 | 0.104 | 0.2332 | Yes |
| 15 | MARK2 |  |  | 738 | 0.103 | 0.2430 | Yes |
| 16 | ASNS |  |  | 850 | 0.094 | 0.2411 | Yes |
| 17 | CDC34 |  |  | 855 | 0.094 | 0.2514 | Yes |
| 18 | WIZ |  |  | 970 | 0.087 | 0.2483 | Yes |
| 19 | ATP6V1C1 |  |  | 1172 | 0.077 | 0.2340 | Yes |
| 20 | ARRB2 |  |  | 1186 | 0.076 | 0.2413 | Yes |
| 21 | SOD2 |  |  | 1228 | 0.075 | 0.2451 | Yes |
| 22 | YKT6 |  |  | 1307 | 0.071 | 0.2443 | Yes |
| 23 | UROD |  |  | 1324 | 0.070 | 0.2506 | Yes |
| 24 | AGO2 |  |  | 1338 | 0.070 | 0.2571 | Yes |
| 25 | SLC6A8 |  |  | 1391 | 0.068 | 0.2589 | Yes |
| 26 | AMD1 |  |  | 1460 | 0.066 | 0.2586 | No |
| 27 | PPP1R2 |  |  | 1724 | 0.056 | 0.2348 | No |
| 28 | EIF5 |  |  | 1749 | 0.055 | 0.2383 | No |
| 29 | CDKN1C |  |  | 1788 | 0.054 | 0.2401 | No |
| 30 | PSMC3 |  |  | 1903 | 0.051 | 0.2328 | No |
| 31 | PDAP1 |  |  | 1999 | 0.048 | 0.2274 | No |
| 32 | BTG3 |  |  | 2012 | 0.047 | 0.2315 | No |
| 33 | BAK1 |  |  | 2122 | 0.044 | 0.2240 | No |
| 34 | CDO1 |  |  | 2145 | 0.043 | 0.2264 | No |
| 35 | STIP1 |  |  | 2150 | 0.043 | 0.2309 | No |
| 36 | PRKACA |  |  | 2177 | 0.042 | 0.2328 | No |
| 37 | ACAA1 |  |  | 2308 | 0.038 | 0.2223 | No |
| 38 | IRF1 |  |  | 2377 | 0.037 | 0.2187 | No |
| 39 | LHX2 |  |  | 2562 | 0.032 | 0.2011 | No |
| 40 | CASP3 |  |  | 2634 | 0.030 | 0.1965 | No |
| 41 | KLHDC3 |  |  | 2649 | 0.030 | 0.1983 | No |
| 42 | MRPL23 |  |  | 2860 | 0.025 | 0.1770 | No |
| 43 | CYB5B |  |  | 3128 | 0.019 | 0.1485 | No |
| 44 | CDC5L |  |  | 3466 | 0.012 | 0.1110 | No |
| 45 | POLE3 |  |  | 3486 | 0.012 | 0.1102 | No |
| 46 | PDLIM3 |  |  | 3503 | 0.011 | 0.1096 | No |
| 47 | SIGMAR1 |  |  | 3673 | 0.007 | 0.0910 | No |
| 48 | STARD3 |  |  | 3993 | 0.001 | 0.0544 | No |
| 49 | ALDOA |  |  | 4006 | 0.001 | 0.0531 | No |
| 50 | CCND3 |  |  | 4016 | 0.000 | 0.0521 | No |
| 51 | JUNB |  |  | 4044 | -0.000 | 0.0490 | No |
| 52 | TYRO3 |  |  | 4085 | -0.001 | 0.0445 | No |
| 53 | MSX1 |  |  | 4097 | -0.001 | 0.0433 | No |
| 54 | NPTXR |  |  | 4153 | -0.002 | 0.0373 | No |
| 55 | NUP58 |  |  | 4204 | -0.003 | 0.0319 | No |
| 56 | DNAJB1 |  |  | 4510 | -0.009 | -0.0022 | No |
| 57 | FOS |  |  | 4536 | -0.010 | -0.0040 | No |
| 58 | DGAT1 |  |  | 4639 | -0.011 | -0.0144 | No |
| 59 | FKBP4 |  |  | 4706 | -0.013 | -0.0206 | No |
| 60 | GRINA |  |  | 4752 | -0.013 | -0.0242 | No |
| 61 | PTPRD |  |  | 4827 | -0.015 | -0.0310 | No |
| 62 | CCNE1 |  |  | 4890 | -0.016 | -0.0363 | No |
| 63 | FURIN |  |  | 4962 | -0.017 | -0.0424 | No |
| 64 | RXRB |  |  | 5013 | -0.019 | -0.0460 | No |
| 65 | TST |  |  | 5025 | -0.019 | -0.0451 | No |
| 66 | RAB27A |  |  | 5032 | -0.019 | -0.0436 | No |
| 67 | POLR2H |  |  | 5073 | -0.020 | -0.0460 | No |
| 68 | CYB5R1 |  |  | 5097 | -0.020 | -0.0463 | No |
| 69 | KCNH2 |  |  | 5165 | -0.022 | -0.0515 | No |
| 70 | CTSV |  |  | 5544 | -0.029 | -0.0916 | No |
| 71 | CLCN2 |  |  | 5554 | -0.030 | -0.0892 | No |
| 72 | ALAS1 |  |  | 5763 | -0.034 | -0.1093 | No |
| 73 | ENO2 |  |  | 5889 | -0.036 | -0.1196 | No |
| 74 | CA2 |  |  | 5950 | -0.037 | -0.1222 | No |
| 75 | PARP2 |  |  | 6131 | -0.041 | -0.1382 | No |
| 76 | AP2S1 |  |  | 6273 | -0.044 | -0.1494 | No |
| 77 | MGAT1 |  |  | 6475 | -0.049 | -0.1669 | No |
| 78 | TFRC |  |  | 6511 | -0.050 | -0.1652 | No |
| 79 | CHKA |  |  | 6526 | -0.050 | -0.1610 | No |
| 80 | E2F5 |  |  | 6554 | -0.052 | -0.1581 | No |
| 81 | DNAJA1 |  |  | 6816 | -0.059 | -0.1814 | No |
| 82 | OLFM1 |  |  | 6897 | -0.062 | -0.1835 | No |
| 83 | EPHX1 |  |  | 7008 | -0.065 | -0.1887 | No |
| 84 | NTRK3 |  |  | 7017 | -0.065 | -0.1821 | No |
| 85 | NFKBIA |  |  | 7027 | -0.066 | -0.1755 | No |
| 86 | PPT1 |  |  | 7033 | -0.066 | -0.1685 | No |
| 87 | HNRNPU |  |  | 7074 | -0.067 | -0.1654 | No |
| 88 | SHOX2 |  |  | 7163 | -0.070 | -0.1675 | No |
| 89 | FEN1 |  |  | 7218 | -0.071 | -0.1655 | No |
| 90 | CHRNA5 |  |  | 7258 | -0.073 | -0.1616 | No |
| 91 | BMP2 |  |  | 7299 | -0.074 | -0.1576 | No |
| 92 | RHOB |  |  | 7306 | -0.075 | -0.1497 | No |
| 93 | PPAT |  |  | 7406 | -0.078 | -0.1522 | No |
| 94 | HSPA13 |  |  | 7471 | -0.080 | -0.1503 | No |
| 95 | SLC25A4 |  |  | 7519 | -0.082 | -0.1462 | No |
| 96 | PRPF3 |  |  | 7702 | -0.091 | -0.1567 | No |
| 97 | BID |  |  | 7709 | -0.092 | -0.1469 | No |
| 98 | EIF2S3 |  |  | 7783 | -0.094 | -0.1444 | No |
| 99 | GLS |  |  | 7856 | -0.099 | -0.1413 | No |
| 100 | GRPEL1 |  |  | 7920 | -0.102 | -0.1368 | No |
| 101 | CDK2 |  |  | 8018 | -0.108 | -0.1355 | No |
| 102 | HYAL2 |  |  | 8088 | -0.113 | -0.1305 | No |
| 103 | DDX21 |  |  | 8172 | -0.119 | -0.1264 | No |
| 104 | TAP1 |  |  | 8393 | -0.146 | -0.1349 | No |
| 105 | IL6ST |  |  | 8394 | -0.146 | -0.1181 | No |
| 106 | BSG |  |  | 8443 | -0.151 | -0.1062 | No |
| 107 | TMBIM6 |  |  | 8673 | -0.225 | -0.1067 | No |
| 108 | GGH |  |  | 8702 | -0.239 | -0.0824 | No |
| 109 | IGFBP2 |  |  | 8719 | -0.260 | -0.0544 | No |
| 110 | RPN1 |  |  | 8736 | -0.272 | -0.0249 | No |
| 111 | CCK |  |  | 8739 | -0.275 | 0.0066 | No |
Table: GSEA details [plain text format]

  

Fig 2: HALLMARK\_UV\_RESPONSE\_UP: Random ES distribution      
 Gene set null distribution of ES for **HALLMARK\_UV\_RESPONSE\_UP**
